# Role of the intestinal microbiome and its therapeutic intervention in cardiovascular disorder

**DOI:** 10.3389/fimmu.2024.1321395

**Published:** 2024-01-26

**Authors:** Ameer Luqman, Adil Hassan, Mehtab Ullah, Sahar Naseem, Mehraj Ullah, Liyuan Zhang, Ahmad Ud Din, Kamran Ullah, Waqar Ahmad, Guixue Wang

**Affiliations:** ^1^ Key Laboratory for Biorheological Science and Technology of Ministry of Education, State and Local Joint Engineering Laboratory for Vascular Implants, Bioengineering College of Chongqing University, Chongqing, China; ^2^ JinFeng Laboratories, Chongqing, China; ^3^ Chongqing Key Laboratory of Nano/Micro Composite Materials and Devices, Chongqing University of Science and Technology, Chongqing, China; ^4^ School of Fermentation Engineering Tianjin University of Science and Technology, Tianjin, China; ^5^ Plants for Human Health Institute, Department of Food, Bioprocessing and Nutrition Sciences, North Carolina State University, Kannapolis, NC, United States; ^6^ Department of Biology, The University of Haripur, Haripur, Khyber Pakhtunkhwa, Pakistan; ^7^ Basic Medicine Research Innovation Center for Cardiometabolic Diseases, Ministry of Education, Southwest Medical University, Luzhou, China

**Keywords:** CVD, HF, HTN, TMAO, SCFAs, FMT

## Abstract

The gut microbiome is a heterogeneous population of microbes comprising viruses, bacteria, fungi, and protozoa. Such a microbiome is essential for sustaining host equilibrium, and its impact on human health can be altered by a variety of factors such as external variables, social behavior, age, nutrition, and genetics. Gut microbes’ imbalances are related to a variety of chronic diseases including cancer, obesity, and digestive disorders. Globally, recent findings show that intestinal microbes have a significant role in the formation of cardiovascular disease (CVD), which is still the primary cause of fatalities. Atherosclerosis, hypertension, diabetes, inflammation, and some inherited variables are all cardiovascular risk variables. However, studies found correlations between metabolism, intestinal flora, and dietary intake. Variations in the diversity of gut microbes and changes in their activity are thought to influence CVD etiology. Furthermore, the gut microbiota acts as an endocrine organ, producing bioactive metabolites such as TMA (trimethylamine)/TMAO (trimethylamine N-oxide), SCFA (short-chain fatty acids), and bile acids, which have a substantial impact on host wellness and disease by multiple mechanisms. The purpose of this overview is to compile current evidence highlighting the intricate links between gut microbiota, metabolites, and the development of CVD. It focuses on how intestinal dysbiosis promotes CVD risk factors such as heart failure, hypertension, and atherosclerosis. This review explores the normal physiology of intestinal microbes and potential techniques for targeting gut bacteria for CVD treatment using various microbial metabolites. It also examines the significance of gut bacteria in disease treatment, including supplements, prebiotics, probiotics, antibiotic therapies, and fecal transplantation, which is an innovative approach to the management of CVD. As a result, gut bacteria and metabolic pathways become increasingly attractive as potential targets for CVD intervention.

## Graphical Abstract

The significance of microbial-mediated metabolites in the emergence of CVD. CVD, cardiovascular disease; SCFAs, short-chain fatty acids; TMA, trimethylamine; TMAO, trimethylamine N-oxide; LPS, lipopolysaccharides.

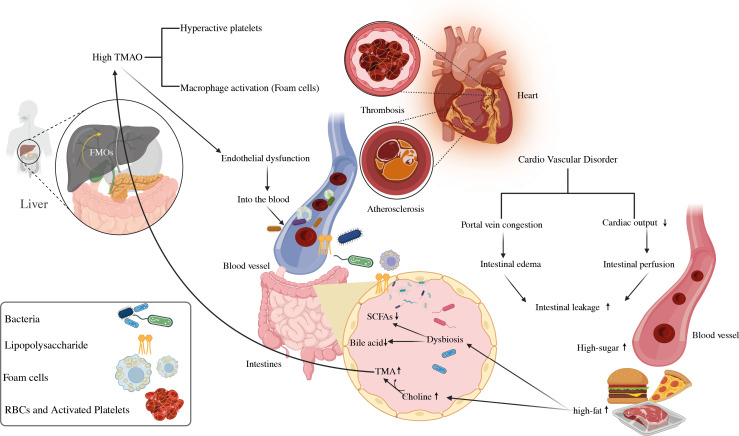

## Introduction

Understanding the evolution of the gut microbiota and its internal and external impacts on the intestine, as well as the risk factors for cardiovascular diseases (CVDs), such as metabolic syndrome, has attracted a great deal of attention (1, 2). Globally, CVDs are the primary causes of death, encompassing conditions including coronary artery disease (CAD), atherosclerosis, thrombosis, aneurysms, arterial hypertension, and cardiomyopathies that reinforce heart failure and cerebrovascular diseases (3–5). The current studies predict 17.5 million deaths per year by CVD, accounting for around 31% of all overall mortality (6). Among them, heart attack and stroke are directly linked to 85% of the cases (7, 8). Inflammation, dyslipidemia (i.e., elevated serum cholesterol, triglycerides, and low-density lipoproteins), and diabetes mellitus are prevalent pathological mechanisms and risk factors that can impact the progression and emergence of CVD (9, 10). In addition to hereditary factors, environmental factors such as nutrition and gut microbiota composition are going to play a significant influence in the development of CVDs. Furthermore, the rise of obesity and diabetes has been related to intestinal dysbiosis (11, 12), insulin resistance, and sedentary behaviors such as smoking, insufficient exercise, and poor nutrition are all identified risk variables for CVD (13, 14). The research into how the human gut microbiome affects CVD and metabolic diseases has expanded dramatically (15). Gut dysbiosis is a condition defined by changes in intestinal bacteria in adults, can be induced by a range of events such as dietary choices, environmental effects, intestinal infections, or external variables, and it can result in inflammation and metabolic disorders (16). The human gut microbiome comprises an array of over 10 trillion diverse microbes, encompassing bacteria, viruses, protozoa, methanogen archaea, and fungi. This collective term, microbiota, is synonymous with the entirety of these microbial inhabitants residing within the human body (17), while *Actinobacteria, Firmicutes, Proteobacteria*, and *Bacteroides* are the four major bacterial genera that comprise a healthy microbiota (18, 19). Before birth, an infant’s gut has very few germs (20), but after birth, the body begins to receive a steady stream of stimulation from the environment. It promotes a gradual rise in the number of bacteria in the colon, eventually leading to the formation of a dynamic and balanced balance in the gut microbiota (21).

**Figure 1:**
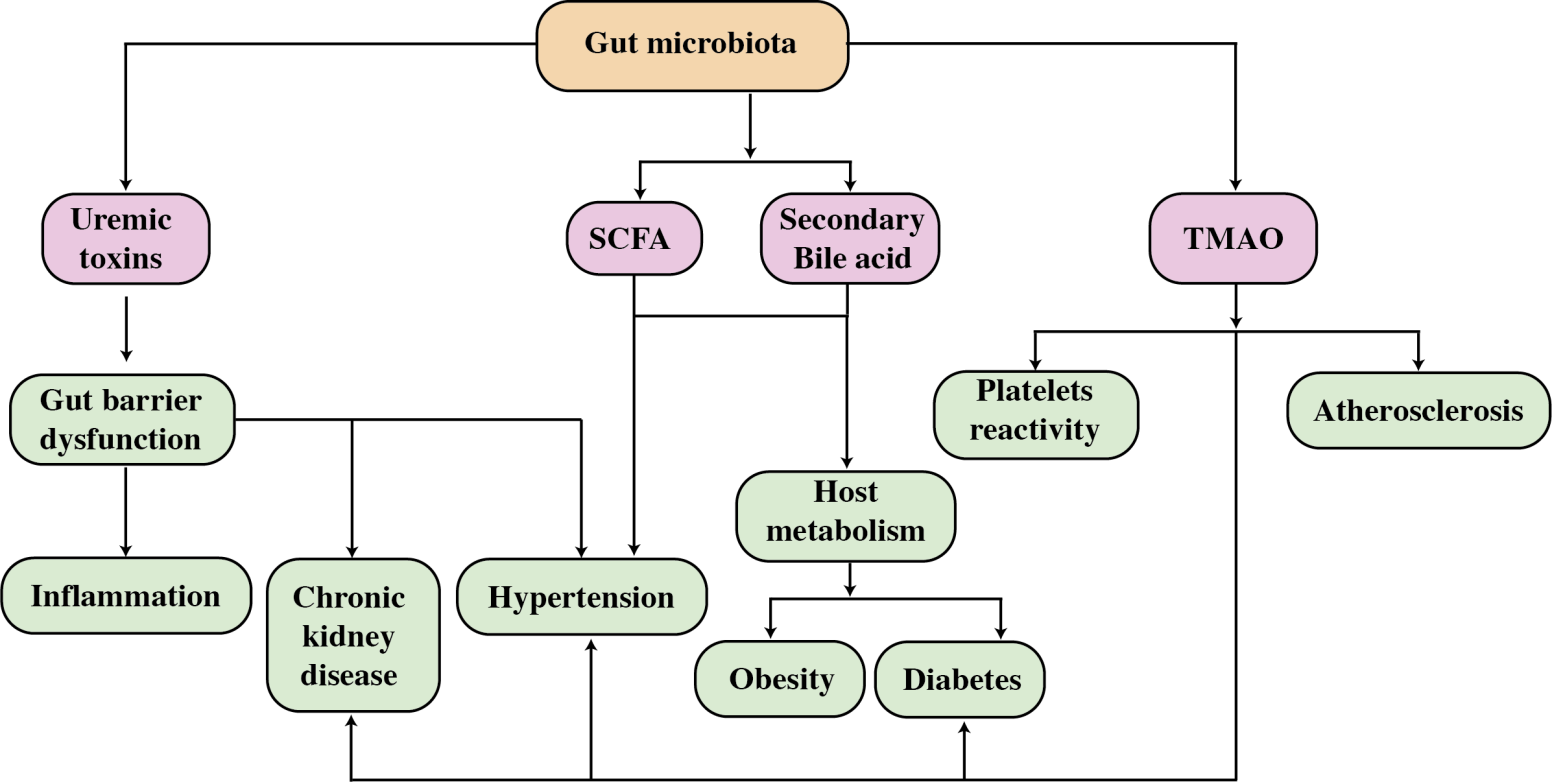
A diagram depicting the effect of gut bacteria and metabolites on CVD risk factors. SCFAs, short-chain fatty acids; TMAO, trimethylamine N-oxide.

The intestinal mucosal surface serves as the interface between the gut microbiota and performs a number of tasks that keep the intestinal epithelial barrier functioning (22). Endotoxins, microbes, and their byproducts can move more easily through the gut wall and into the bloodstream, where they can cause autoimmune disorders. Immune dysregulation and inflammation are at the basis of many CVDs, including atherosclerosis, myocardial infarction (MI), rhythm disorders, pericardial disease, cardiomyopathies, and heart failure (23). Moreover, it is important to highlight that the intestinal tract can be seen as an extensive and diverse ecosystem that produces a significant number of microbial metabolites (24). The host food is broken down by the gut flora into a variety of metabolically active products, including trimethylamine N-Oxide (TMAO), short-chain fatty acids (SCFAs), primary and secondary bile acids, tryptophan and indole derivatives, phenylacetylglutamine (PAGln), and branched-chain amino acids (BCAA), these may contribute to the CVD progression (25). Moreover, N-oxide TMA/TMAO and bile acids are fascinating biomarkers for CVD progression (26), however other gut microbiota components or similar chemicals should be investigated for use as early CVD markers (11). The loss of SCFA-producing intestinal microbes would disrupt the equilibrium of glucose metabolism, raising the risk of CVD (27). Trimethylamine stimulates macrophage stimulation, producing vascular damage; excessive TMAO levels owing to intestinal dysbiosis promote atherosclerosis, raise the risk of CAD, and hasten arterial plaque development that leads to cardiovascular disease (6). As shown in **Figure 1**, reducing dietary TMAO precursor intake is a promising strategy for lowering the risk of CVD due to the high amounts of trimethylamine (TMA) and TMAO generation by choline-induced gut flora (28, 29). Microbial sequencing analysis has emerged as a valuable tool for uncovering distinct gut microbiota patterns linked to cardiovascular disease CVD (30–32).

The gut microbiota plays a crucial role in influencing overall health, either through direct mechanisms or indirect pathways. The intricate interactions involving variations in microbiome composition, metabolites, and CVD susceptibility underscore the significance of intestinal microbes as a novel modulator of CVD. The identified association between gut microbes and CVD suggests that modifying the intestinal microbiota could be beneficial in preventing and managing the development of CVD. Nutritional therapy, the use of pre/probiotics and antibiotics, fecal microbiota transplantation, TMAO reduction, and regular exercise are all current ways to manage gut bacteria to improve cardiovascular function (11). The latest study highlights the possible importance of microbial imbalance in CVD disorders. The advent of genomic and metabolomic technologies has allowed for more thorough characterization and molecular research of these microbiota and their metabolites. However, most evidence continues to indicate associations and the particular chemical processes driving a majority of visible events remain unidentified (33). Future studies focusing on microbe-microbe and microbe-host interactions could reveal how specific metabolites influence the disease process. It is also critical to have a better understanding of the bacterial mechanisms involved in the production of CVD-related metabolites, and also their functional roles. These results could provide a solid theoretical basis for the invention of therapeutic methods for CVD individuals. The present paper covers the usual composition and functional significance of intestinal bacteria and also provides new insights into the gut microbiota and its linked metabolites, which are implicated in CVDs. Scientific studies, putative biological explanations, and therapeutic outcomes are of significant interest to researchers. In addition, we discuss studies relating the gut microbiota to inflammatory processes, lipid metabolic disorders, and diabetes, all of which are linked to an elevated risk of cardiovascular disease. As a result, this overview focuses primarily on studying the role of gut microbiota-related metabolites and their therapeutic potential in CVDs, which may eventually provide more insight into the development of CVD prevention.

## Gut microbiota and TMAO metabolite

The intestinal *Bacteroidetes* are one of the most significant bacterial colonies in the gastrointestinal microbiota. Despite their wide species composition, these cultures display stability in many gut regions, and some exhibit location-specific differentiation, mainly in the ascending colon (34). A total of around 1,000 different species of intestinal microbes, comprising about 10^14^, and bacterial-to-human cell ratio varied between 10:1 and 1:1 (35). In cardiovascular patients, more than 90% of these bacteria had an impact on the growth of *Bacteroidetes* and *Firmicutes*, keeping a stable *Firmicutes/Bacteroidetes* (F/B) ratio (36). The emergence of CVD is dependent on a compromised mucosal barrier and decreased intestinal mucosal barrier function, and is mostly caused by gram-negative microbes, such as lipopolysaccharide (LPS), which plays a significant role in the emergence of cardiometabolic diseases (37, 38). A high-fat diet has been shown to reduce gram-positive *Bifidobacteria* levels in the digestive system while increasing the amount of intestinal microbes that hold LPS, both of which contribute to obesity, the primary risk factor for CVD (39). The F/B ratio become a crucial role in the context of obesity, particularly in children (40). This ratio is linked to low-grade inflammation, which increases the probability of diabetes, a known risk factor for CVDs (41).

**Figure 2:**
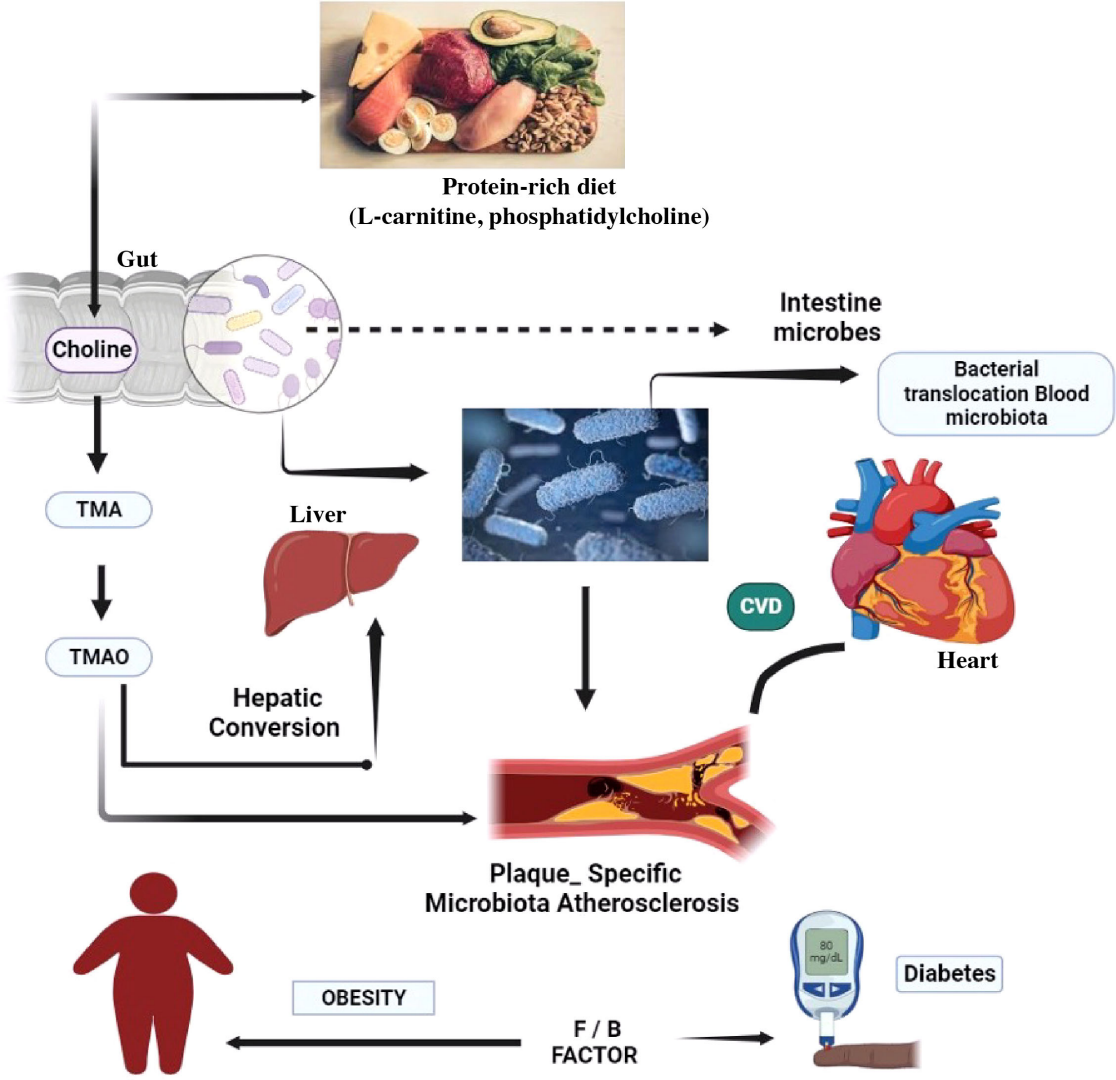
The gut microbiota of the target body’s functioning mechanisms. A low-fiber diet corresponds with decreased short-chain fatty acid butyrate formation, exacerbating dysbiosis and sustaining local and systemic inflammation via bacterial toxin leaks, most notably LPS. A modern Western diet strong in red meat promotes the synthesis of TMA by bacteria, which is then oxidized in the liver to the pro-atherosclerotic metabolite. CVD, cardiovascular disease; TMA, trimethylamine; TMAO, trimethylamine N-oxide.

Because the gut acts as a bridge between them, the interaction between the host and the gut microbiota is crucial for preserving intestinal integrity. Several microbial metabolites have been linked to CVD (25) including bile acids, SCFAs, branched-chain amino acids, TMAO, tryptophan, and indole derivatives (42). The TMAO is formed when foods rich in choline, lecithin, and L-carnitine, primarily found in animal products, with limited plant-based sources, are ingested. In the gut, lecithin (comprising phosphatidylcholine, a choline source) and dietary choline are metabolized into TMA by the gut microbiota having specialized enzymes TMA lyses transcribed by cutC/cutD genes found in various bacterial strains. Recent data suggests that elevated circulating levels of TMAO are associated with an increased risk of CVD and mortality (43–46). Increased TMAO levels in the bloodstream encourage lipid accumulation in the arteries, which contributes to atherosclerosis. **Figure 2** depicts how the inflammatory response influences the development of glucose intolerance, diabetes, and CVD (47–50). A dysbiotic microbe was found to decrease the amount of cholesterol eliminated by feces while increasing absorption and plasma levels of low-density lipoproteins, signaling that dysbiosis may increase the risk of atherosclerosis and CVD (39).

### Gut microbiota composition, diversity, and risk factors

**Figure 3:**
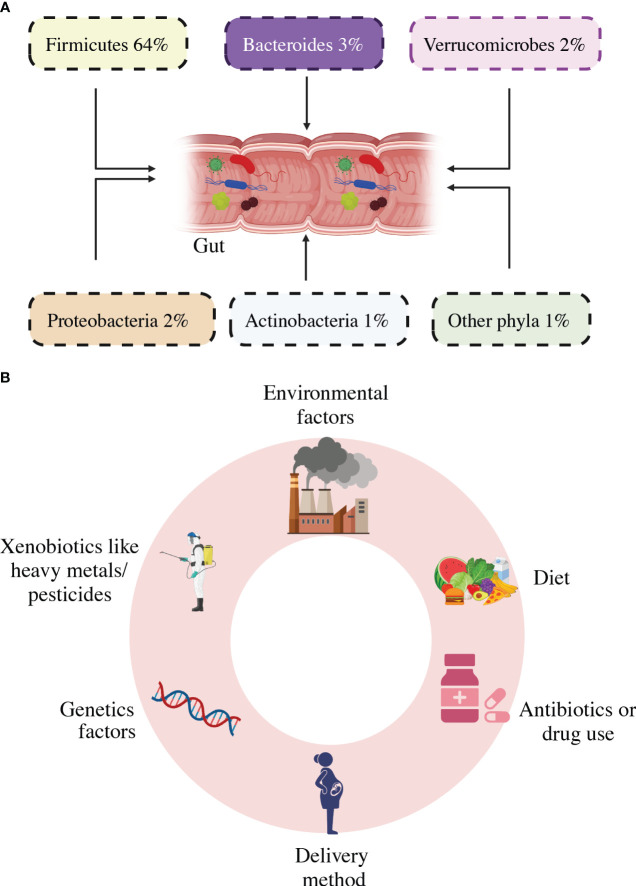
Gut microbiota composition **(A)** and diversity and Dysbiosis risk factors **(B)**.

The bacterial composition, diversity, and abundance are highly influenced by genetic changes in the host’s genome, and by external variables such as the host’s lifestyle, diet, sanitation, health, and the use of antibiotics and probiotics (51). The gut microbiota has small genetic differences in various parts of the intestine. Eckburg et al., (24) used metagenomic analysis to discover the gut bacterial community is made up of six phyla: *Firmicutes, Bacteroidetes, Proteobacteria, Actinobacteria, Fusobacteria*, and *Verrucomicrobia*, with the majority of the organisms in a healthy bacterial community being anaerobic population, as shown in **Figure 3** (52). They inhabit unique biological niches on mucosal surfaces and in the gut lumen, where they form sophisticated biochemical interaction networks with both their hosts and with others (53). The synergistic interaction between the host species and the gut microbiome fosters the proliferation of beneficial microbes while inhibiting the growth of harmful bacteria (54). The gut flora regulates many bodily functions, such as providing metabolic fuel to the host, supporting growth and immune system regulation, removing harmful microbes, keeping intestinal wall integrity, and maintaining overall homeostasis (55). Microbial life has a significant impact on immune function and metabolism, with well-balanced gut microbiota playing an important role in the health of the host. (56). Inadequate dietary intake, excessive stress, significant life events, and antibiotic administration can all impact the diversity of the gut microbiota, leading to a disorder called dysbiosis (20). Elie Metchnikoff, a Russian immunologist and microbiologist renowned for his contributions to the understanding of the immune system, particularly the concept of phagocytosis, is not credited with coining the term “dysbiosis” (57, 58). An imbalance in the typical microbial composition (microbiota) of the colon or other bodily regions is described by this term, which is used in the current discipline of microbiology (59). In particular, Metchnikoff’s study has little work with the current concept of dysbiosis, which emerged as knowledge of the function of the human microbiome in health has increased (60). As seen in **Figure 3**, several risk factors were put out as potential causes of intestinal dysbiosis. There is a lot of literature known about the use of antibiotics, which has been seen to alter the composition of the gut’s microbiome and have both short-term and long-term effects (61–64). Obesity and high-fat and sugar meals are all related to persistent variations in the gut microbiota (65–68).

It is believed that external factors at various stages of life influence the formation of gut dysbiosis. The style of delivery, type of feeding, and hospital milieu are all related to the diversity of the bacteria during childhood (69, 70). Further, social stresses and exposure to xenobiotics including pesticides and heavy metals have been related to gut dysbiosis (71, 72). The emergence of a gut microbiome has a genetic basis as opposed to social factors, based on twin studies. Given that identical twins have nearly identical DNA, any changes in their gut microbes must be the result of non-genetic variables like food, medical history, or use of antibiotics (73, 74). The study of bacterial genomes has transformed the field of microbial research. Metagenomic sequencing and 16S rDNA sequencing are two types of sequencing that are frequently utilized to assess the abundance of microbial components. By focusing on the conserved sections that surround the hypervariable regions, the 16S sequencing approach can detect variations in bacterial genomes (75, 76).

## Impact of gut microbiota on cardiovascular disease

**Figure 4:**
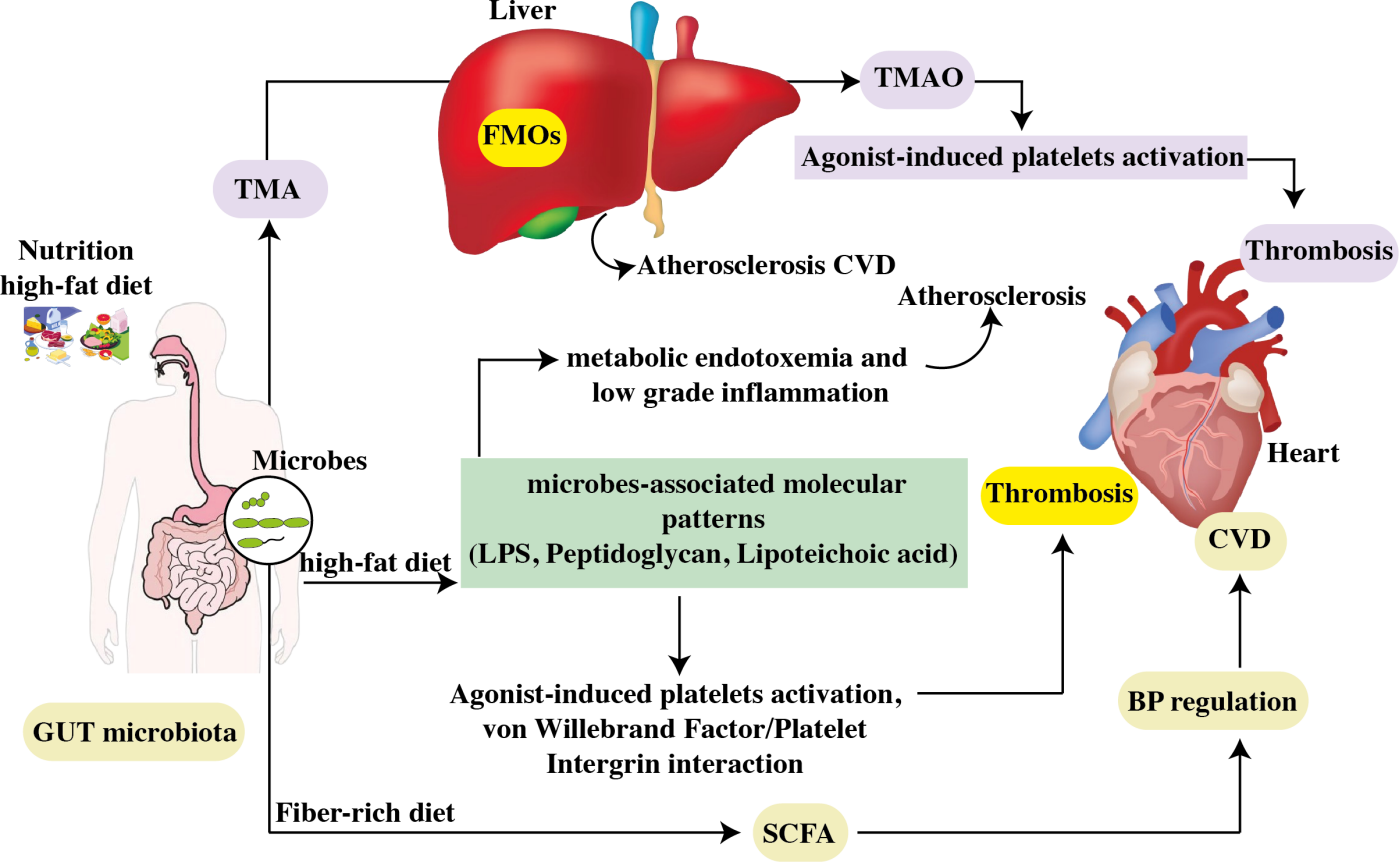
The contribution of the gastrointestinal microbiome to CVD. Choline, phosphatidylcholine, and carnitine are all available in high-cholesterol, high-fat diets. Intestinal microbes convert phosphatidylcholine in the diet to choline, which is then turned into trimethylamine. Hepatic flavin monooxygenases convert TMA to TMAO in the liver. By increasing atherosclerosis and generating agonist-induced platelet activation, TMAO promotes thrombosis. High levels of TMAO in the blood are linked to an increased risk of CVD. Furthermore, a high-fat diet raises the levels of microbe-associated molecular patterns like LPS. Increased intestinal uptake of microbe-associated molecular patterns results in metabolic endotoxemia and low-grade inflammation, both of which worsen atherosclerosis. TLR2 induces arterial thrombosis by increasing the interaction between von Willebrand factor and platelet integrin. Furthermore, intestinal bacteria convert carbs to SCFA and produced by gut microbial fermentation regulate blood pressure, a risk factor for CVD progression. FMO: flavin monooxygenases, SCFAs, short-chain fatty acids; TMAO, trimethylamine N-oxide; TLR2, toll-like receptor-2; LPS, lipopolysaccharide.

A broad spectrum of diseases is considered in CVD, including atherosclerosis, aortic valve disease, peripheral artery disease, hypertension, and stroke. Heart failure, hypertension, and atherosclerosis are associated with gut dysbiosis as shown in **Table 1**. In recent years, significant progress has been made in understanding how the gut microbiome impacts cardiovascular function and the development of these diseases. In this specific section, we intend to highlight several well-supported studies that deliver compelling evidence regarding the role of gut microbiota in the development of cardiovascular disease as depicted in **Figure 4** (92).

**Table 1:** Modifications in the diversity of the intestinal microbes attributed to CVD. CVD-related changes in the diversity of the gut’s microbes.

Species	Technique	Modifications in gut microbial diversity attributed to diseases		References
		Decrease	Increase	
Atherosclerosis and coronary artery disease				
Human	Metagenomics sequencing	*Bactericides* and *Prevotella*	*Streptococcus* and *Escherichia*	(77, 78)
Human	Terminal restriction fragment length polymorphism	*Bactericides* and *Prevotella*	Order *Lactobacillales*	(79)
Human	Metagenomics sequencing	*Roseburia* and *Eubacterium*	*Collinsella*	(31, 33)
Human	16S sequencing	*Clostridium, Faecalibacterium*	*Prevotella*	(80)
Human	16S sequencing	*Burkholderia, Corynebacterium* and *Sediminibacterium*, *Comamonadaceae, Oxalobacteraceae, Rhodospirillaceae, Bradyrhizobiaceae* and *Burkholderiaceae*	*Curvibacter*, *Burkholderiales, Propionibacterium, Ralstonia*	(33, 81)
Hypertension				
Human	Metagenomic sequencing		*Prevotella* and *Klebsiella*	(82)
Human	Metagenomic sequencing	*Roseburia* spp., *Faecalibacterium prausnitzii*,	*Klebsiella* spp., *Streptococcus* spp., and *Parabacteroides merdae*	(83)
Human	16S sequencing	Butyrate-producing bacteria *Odoribacter*		(84, 85)
Heart failure				
Human	16S sequencing	*Blautia, Collinsella, uncl. Erysipelotrichaceae* and *uncl. Ruminococcaceae*		(86, 87)
Human	Incubation with a selective agar		*Campylobacter, Shigella, Salmonella, Yersinia Enterocolitica*,	(88)
Human	16S sequencing	*Faecalibacterium*	*Lactobacillus*	(89)
Human	Metagenomic sequencing	*Faecalibacterium prausnitzii*	*Ruminococcusgnavus*	(90)
Atrial fibrillation				
Human	Metagenomic sequencing	*Faecalibacterium, Alistipes, Oscillibacter*, and *Bilophila*	*Ruminococcus, Streptococcus*, and *Enterococcus*,	(91)

### Gut bacteria; heart failure (HF) with microbial metabolites and current treatments

The disease termed heart failure is defined by the heart’s decreased ability to efficiently pump enough blood and oxygen to satisfy the demands of the body (93, 94). It serves as the final stage of multiple CVDs, which are highly prevalent, have significant mortality rates, and pose a significant threat to human well-being (95). While chronic exposure is defined by an altered inflammatory state related to pro-inflammatory aspects that are critical to the beginning of HF, immediate exposure is associated with a variety of inflammation-related symptoms (96). Our understanding of the pathophysiological processes behind HF has greatly increased. The recognition of the vital role of managing neurohumoral processes rather than focusing solely on changes in blood flow is a key shift in this understanding (97, 98). More evidence indicates the stomach is implicated in decreased heart rate and higher systemic congestion, both of which can contribute to intestinal mucosal ischemia and edema. As a result, bacterial translocation may be enhanced, allowing endotoxins into the bloodstream and contributing to the inflammation seen in HF patients (99). Niebauer et al. (100) conducted a study that revealed a significant association between peripheral edema in HF patients and higher plasma levels of endotoxins and inflammatory cytokines. Specifically, patients experiencing peripheral edema demonstrated elevated concentrations of these markers compared to those without edema. However, the study found a decrease in serum amounts of endotoxins but not cytokines following short-term diuretic treatment. This finding raises the prospect that edema and gut-associated inflammation in cardiac failure may be linked while diuretic medication might be effective (100). Pasini et al. (88) showed a recent comparison of the bacterial and fungal profiles in the feces of heart failure patients and the findings revealed people with chronic heart failure (CHF) had a greater risk of perilous bacterial growth compared with control group.

The presence of *Candida*, *Campylobacter*, and *Shigella* species was linked to 78.3% of CHF disease severity that had significantly greater intestinal permeability. Between TMAO and the risk of atherosclerosis, there was a strong positive correlation between gut permeability and right atrial pressure. An increased TMAO levels have been linked to poor outcomes in people with heart failure (101, 102). Scientists evaluated the levels of TMAO in 2,490 patients with chronic heart disease in the study with a 9.7-year follow-up. The data showed increasing TMAO levels coincided with increased rates of morbidity and mortality, particularly in HFrEF patients. This study suggests that TMAO could be utilized as a biomarker to predict poor outcomes in HfrEF patients (103). A recent meta-analysis offered that some reliable insights into the prognostic importance of TMAO in HF (102). The higher TMAO precursor trimethyl lysine (TML)-derived N, N, N-trimethyl-5-aminovaleric acid (TMAVA) synthesis by the gut microbiota was linked to a progressive reduction in fatty acid oxidation (104). Several studies have consistently reported that patients with HF exhibited a decrease in butyrate-producing bacteria, particularly within the *Lachnospiracea*and *Ruminococcaceae* families (105). However, the absence of butyrate-producing microbes such *Eubacterium Halli* and *Lachnospiracea* is associated with higher mortality, increased inflammation, and severity of disease. This association implies that the abundance of these beneficial gut bacteria may have a major impact on the progress and results of cardiac failure (106). Dysbiosis has been consistently linked to reduced butyrate production in various heart failure cohorts (105). Additionally, bile acids, particularly secondary bile acids produced through the transformation by gut microbiota, play a crucial role in heart failure. Research has indicated a rise in secondary bile acids among individuals with CHF(97). Indoxyl sulfate produced by gut microbial metabolism, has also been linked to cardiac fibrosis and ventricular remodeling. These findings underscore the importance of the gut microbiota and its metabolites in heart health and offer possible therapeutic targets for heart failure management (107).

### Atherosclerosis and therapeutic options

Atherosclerotic cardiovascular disease is a persistent inflammatory state primarily impacting sizeable and intermediate arteries. Numerous firmly established factors are correlated with atherosclerosis, including hypertension, dyslipidemia, advanced age, and smoking (108). This is characterized by the accumulation of low-density lipoprotein within the artery walls, leading to the formation of atheroma and distinct plaques consisting of proliferative fibrous tissue and calcifications (109, 110). Recent decades, there has been a growing interest in finding out how the gut microbiota plays a significant role in the emergence of atherosclerotic lesions (111). Koren et al. did an analysis using shotgun DNA sequencing focused on the gut metagenome that indicated significant changes in the diversity of gut microbial populations between patients with symptomatic atherosclerosis and those assumed to be healthy controls. These findings strongly suggest that the gut microbiota may play a significant role in the atherosclerosis (30, 31). Further, extensive metagenome-wide association research done on a cohort of 218 atherosclerosis patients and 187 healthy controls confirmed a link between the diseases with changed gut microbiome composition. In particular, the study found that people with atherosclerosis had significantly higher concentrations of *Enterobacteriaceae, Ruminococcusgnavus*, and *Eggerthellalenta* (112, 113). Introducing prebiotics and probiotic strains which enhance the production of SCFA and boost the diversity of beneficial microbes might be a valuable strategy in atherosclerosis prevention strategies (114). Various animal studies, including the work by Chan et al., (115) have explored the impact of probiotics and telmisartan on mitigating atherosclerosis induced by a high-fat diet, resulted in an increase in the *Firmicutes* to *Bacteroidetes* ratio. A diet rich in fats was found to decrease the prevalence of *Eubacterium, Anaeroplasma, Oscillospira, Roseburia*, and *Dehalobacterium*, while simultaneously elevating the quantities of *Allobaculum, Clostridium, Lactobacillus*, and *Bifidobacteria* (116). The findings show *B. fragilis* can produce extracellular vesicles (EVs), which are lipid bilayer particles. Human research has revealed a relationship between infectious and non-infectious disorders, as well as changes in the systemic levels of EVs derived from gut bacteria (117). It was identified as an essential cell-cell communicator with the potential to increase the knowledge of atherosclerotic disease, ranging from biomarkers to disease pathogenesis (118). Proteomic study has revealed unique protein compositions for EV subtypes, with some indicators assisting in the differentiation of EVs via biogenesis processes. Endosomal sorting complexes required for transport (ESCRT) proteins, Alix, and tetraspanins, for example, are exosome markers, whereas ectosome markers include Annexin A2/A5, ARF6, and Enolase 1 (119). EVs are involved in the immunological response, vascular remodeling, endothelial dysfunction, and apoptosis, which all contribute to atherosclerosis, and EVs in plasma may be useful as atherosclerosis indicators (120).

Scientists used the terminal restriction fragment length polymorphism technique for insight into the gut bacteria contents with coronary artery patients. Their research showed that the microbial diversity in the individuals studied experienced unique alterations. Notably, the levels of *Bacteroides* were decreased while the abundance of *Lactobacillales* and *Clostridium subcluster XIVa* increased in the fecal samples of people. These findings suggest the gut microbiota may contribute to the progression of coronary artery disease. Novel strategies for treating or preventing cardiovascular diseases may be developed as a result of a better understanding of such microbial modification (79). A significant negative association found between the decrease in (1) *Eubacterium* and the rise of inflammatory cytokines like matrix metalloproteinase-9 (MMP-9) and E-selectin; (2) Dehalobacterium and adipocyte fatty acid binding protein (A-FABP); and (3) *Roseburia* and MMP-9. This confirms a connection between imbalanced gut microbiota and the development of atherosclerosis (115). Similarly, Stepankova showed experiments signifying the beneficial impact of gut microbiota in inhibiting the atherosclerotic lesions progression (121).The aortas of the germ-free mice fed a low-cholesterol diet showed atherosclerotic plaques. The results of the study offer strong evidence that microorganisms suppress the progress of atherosclerosis (122).

On the contrary, systems depending on metabolism can be affected by gut dysbiosis, which alters a wide variety of metabolites and may have an effect on the onset and development of atherosclerosis (6). One of the several metabolites produced by the gut bacteria, TMAO, plays a crucial role in the formation of atherosclerosis (123). The accumulation of TMAO in the body has been related to an increase in the risk of atherosclerosis and cardiovascular diseases (124). The blood plasma levels of TMAO in mice with normal gut microbiota grew as they were fed a diet high in choline. In contrast, animals given the same choline-rich diet and antibiotic treatment, which changed their gut microbiota, had minimal TMAO amount (43). Compared to the control TMAO levels, the mice with greater TMAO levels displayed a higher amount of foam cell formation and the development of atherosclerotic plaques. The risk of sudden cardiovascular events is increased by TMAO linked to plaque vulnerability to its involvement in developing atherosclerosis (28, 47). High levels of TMAO in the bloodstream have been associated with conditions such as obesity, Type 2 diabetes mellitus, chronic kidney disease (CKD), and CVD (125, 126). Moreover, numerous other studies have shown that SCFAs may have a positive impact on atherosclerosis by suppressing inflammation (127, 128). As a result, SCFAs may reduce cholesterol levels and stop the host from developing lipid deposits. Dyslipidemia can result from decreased SCFA production, whereas probiotics (*Lactobicili*) are effective in reducing cholesterol. According to Dieck et al., (129) probiotic anti-cholesterolemic effect can be induced via bile salt hydrolysis (BSH), interference with hepatic *de novo* lipid synthesis by regulation of SCFA, or satiety hormones. It indicates that SCFAs may have a preventive effect by reducing the risk factors for cardiac disease (130).

### Association of gut microbiome with hypertension (HTN)

Hypertension is a significant worldwide public health concern and stands as the foremost risk factor for cardiovascular diseases, leading to a substantial economic burden on society. Its epidemiology is defined by a high prevalence, notable levels of disability and mortality, and often insufficient awareness (131, 132). As of 2021, it was estimated that around 330 million people in China were affected by cardiovascular diseases, with approximately 245 million individuals having been diagnosed with hypertension (133). The causes of hypertension involve a combination of factors, including genetic predisposition, lifestyle choices, environmental influences, hormonal imbalances, inflammatory processes, and changes in hemodynamic mechanisms (134). The American College of Cardiology, the American Heart Association, and the European Society of Hypertension have collaboratively formulated behavioral guidelines aimed at maintaining optimal blood pressure levels, with a particular emphasis on non-pharmacological strategies (135). These include using the dietary methods to stop hypertension (DASH) diet, which highlights a high intake of fruits and vegetables while minimizing fat consumption, increasing physical activity through specific aerobic exercises, slicing off salt and alcohol consumption, losing weight, and increasing salt and alcohol consumption (136–138).

A small number of studies mostly in animal models have shown an explicit link between gut microbiota and the control of blood pressure (139–142). For instance, Yang et al. (140) conducted a study where they investigated changes in the fecal microbiota of animal models with hypertension, specifically comparing alterations in the spontaneously hypertensive rat and chronic angiotensin II infusion rat models. They observed a notable gut dysbiosis in hypertensive animals characterized by a decrease in microbial richness, diversity, and consistency (140). Kim (143) also found that among hypertension patients, the presence of butyrate-producing bacteria, such as *Butyricimonas* and anaerobic *Corynebacterium*, had significantly decreased. Studies have revealed a positive correlation between blood pressure and the levels of *Ruminococcaceae*, *Streptococcus*, and *Turicibacter* (140, 144, 145). The metabolite production derived from microbes may also be impacted by changes in gut microbiota. Since they are created by bacterial digestion of dietary fiber and are closely related to good health, SCFAs are of particular significance among these metabolites that are formed from microbes (146), play a vital role in the HTN development. A larger amount of research indicates the potential of SCFAs may effectively decrease the host’s blood pressure by interacting with G protein-coupled receptor 41 (147–149). Although TMAO is required for disease start, an animal model was first used to demonstrate the relationship between TMAO and CVD in 2011 (28). Recently, Wang et al. (150) have provided compelling evidence of a causal link between TMAO and its precursors with blood pressure by employing a Mendelian Randomization approach. Moreover, multiple studies have validated the strong association between elevated TMAO levels and an increased prevalence of hypertension (151–153). Ge et al. (153) proved that a rise of 5 and 10 mol/L in TMAO levels corresponded to a 9% and 20% escalation in the risk of hypertension, respectively. Apart from TMAO, other gut microbiota-derived metabolites, including corticosterone, H2S, choline, BAs, indole sulfate, and LPS, are also produced. The SCFAs, TMAO, BAs, H2S, and LPS metabolites have been closely linked to the development of hypertension. So, the intestinal microbiota may have an interconnected role in regulating blood pressure, and any disruptions in their function could be linked to hypertension. Studies have proposed that *Lactobacillus* probiotics might play a beneficial role in blood pressure regulation (154) Additionally, a meta-analysis has shown that probiotics treatment can lead to a significant reduction in blood pressure in patients (155).

## Role of microbial derived metabolites and CVD

**Figure 5:**
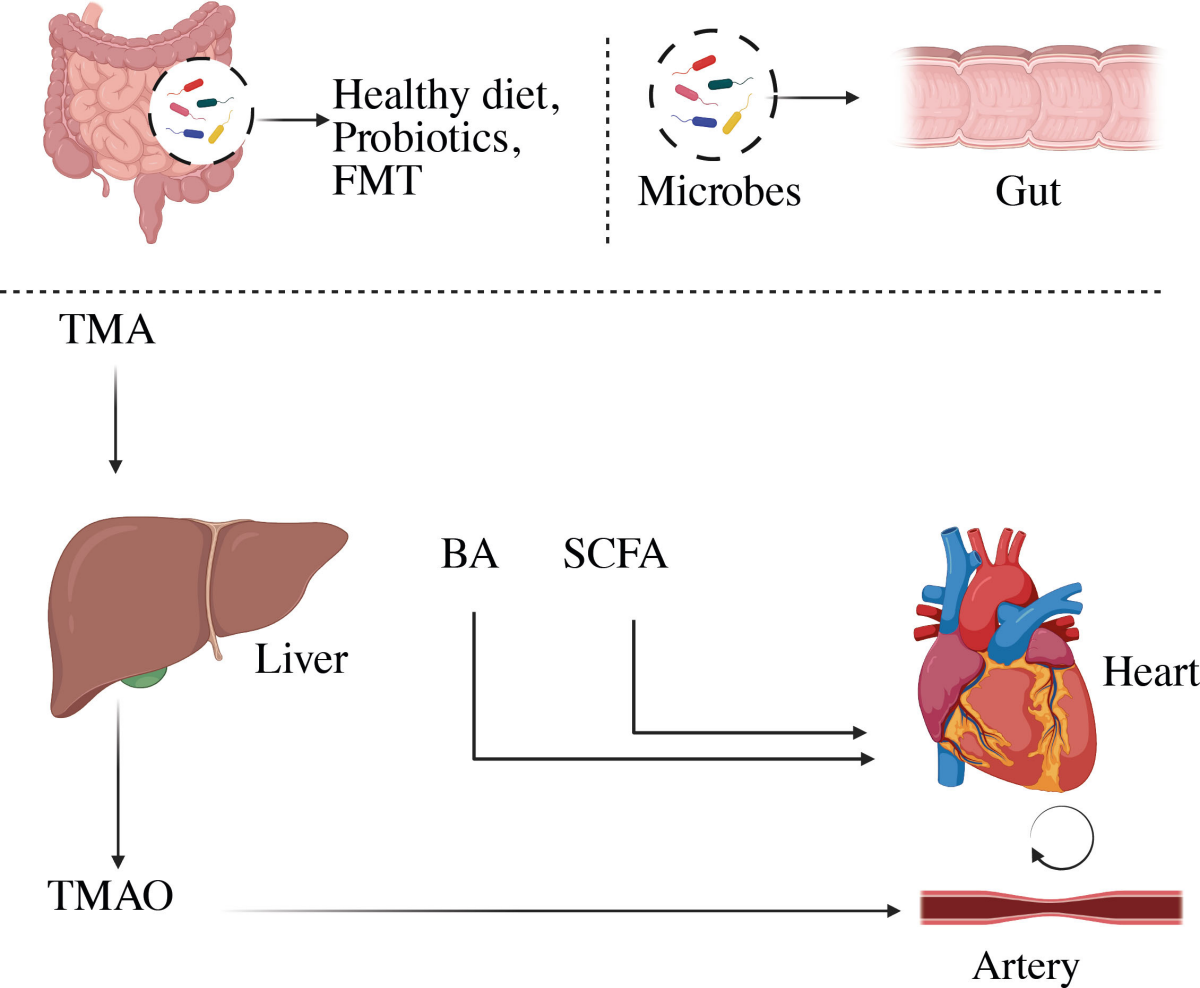
Representation of microbial-derived metabolites to CVD. Variations in the composition of the gut microbiome can change the metabolism, allowing bacteria or its fragments and metabolites to enter the circulation more easily. This can aggravate the pro-inflammatory milieu and produce metabolic disturbances, which can lead to CVD. BA, Bile acid; SCFA, short-chain fatty acids; TMA, trimethylamine; TMAO, trimethylamine-*N*-oxide.

We will briefly discuss the association between trimethylamine N-Oxide and CVD, and focus on other microbial metabolites in this review as illustrated in **Figure 5** (156).

### Trimethylamine-N-oxide (TMAO) associated with CVD

The gut microbial digestion of phosphatidylcholine, the main dietary source of choline, was found to produce a proatherogenic metabolite called trimethylamine-N-oxide (157). Among the numerous physiologically active metabolites of microbial metabolism, TMAO is a biologically active molecule that has been linked to an increased risk of adverse cardiovascular events, including acute coronary syndrome (ACS), stroke, and mortality (47, 158, 159). TMAO production occurs secondary to the ingestion of nutrients containing the trimethylamine moiety, such as choline, phosphatidylcholine, and L-carnitine, all of which are found in high concentrations in animal products, including red meat, fish, milk, and eggs. The metabolism of these nutrients by microbial TMA lyases produces TMA, which enters the portal circulation, is oxidized to TMAO by hepatic flavin monooxygenases, primarily FMO3 (160), and subsequently enters the general circulation (28, 161). It is believed that TMAO may contribute to the development of atherosclerosis, following a proatherogenic pathway. Elevated levels of TMAO in the bloodstream have been positively associated with early atherosclerosis in humans. Moreover, monitoring TMAO levels can be useful in predicting the risk of mortality in patients with stable coronary artery disease and acute coronary syndrome (151, 162). Research has indicated that higher TMAO levels in the bloodstream are linked to the severity of peripheral artery disease and a greater risk of cardiovascular mortality among individuals affected by this condition (163).

In-depth analyses, including meta-analysis and dose-response studies, have further revealed that elevated plasma TMAO levels are associated with a higher occurrence of major adverse cardiovascular events in patients with coronary heart disease (164). Additionally, proinflammatory monocytes and elevated TMAO levels were substantially associated in stroke patients. According to Haghikia et al. (165) higher cardiovascular events such as myocardial infarction, recurrent stroke, and cardiovascular death were also linked to a raised TMAO plasma level. Numerous human investigations have also supported the involvement of TMAO in CVD. Compared to controls, patients with chronic heart failure had higher plasma levels of TMAO, choline, and betaine in a prospective observational analysis of stable CAD and healthy people (166). Similarly, in patients who experienced a myocardial infarction, TMAO was identified as an independent predictor of mortality at the two-year follow-up. The ratio stood at 1.21 (with a 95% confidence interval of 1.03-1.43, P = 0.023), as observed in a study involving 292 events (167). Another study conducted by Tang et al. (48), observed a correlation between elevated TMAO levels and a higher risk of major adverse cardiac events. However, the precise mechanisms by which TMAO affects cardiovascular disease have not been fully investigated.

### Short-chain fatty acids and CVD and prevention strategies

The human digestive system cannot break down complex carbohydrates, such as dietary fiber, to support cell activity. Nevertheless, the gut microbiota can utilize fibers by fermenting them, resulting in the production of SCFAs (168). SCFAs are saturated fatty acids composed of carbon chains ranging from one to six carbons. Acetate, propionate, and butyrate are the main types of SCFAs found in the human body (169). The primary bacteria responsible for producing SCFAs are found in the clostridial clusters IV and XIVa within the *Firmicutes* phylum and include various species of bacteria such as *Eubacterium, Roseburia, Faecalibacterium*, and *Coprococcus* (170). It plays crucial roles in regulating anti-inflammatory responses, lipid metabolism, and gluconeogenesis. Notably, butyrate, one of the SCFAs, is considered a significant energy source for intestinal epithelial cells (171). A significant amount of research shows that SCFAs protect against heart failure and are essential for preserving the integrity of the intestinal barrier by encouraging mucus formation and reducing inflammation (172). The presence of high SCFA levels in fecal samples is linked to markers of hypertension, central obesity, and subclinical indicators of cardiometabolic disorders (173) and to the development of atherosclerosis (174).

Butyric acid in the diet effectively slowed the progression of atherosclerotic plaques in the arterial walls of mice missing apolipoprotein E (Apo-E) in a trial utilizing rodent as a model. The favorable benefits were obtained by slowing macrophage migration, boosting collagen deposition, and improving plaque stability (175). Multiple studies show that the SCFAs contribute to manage blood pressure. For example, when fecal material from hypertension human donors was introduced into germ-free mice vs normotensive donors, researchers saw an increase in blood pressure (176). SCFAs are linked to blood pressure regulation through G-protein coupled receptor (GPCR) pathways, specifically in renin secretion and blood control. SCFA activation of the olfactory receptor (Olfr) 78 and the free fatty acid receptor GPR41 causes an increase in blood pressure and a decrease in blood pressure, respectively. The acetate and propionate have antihypertensive properties due to their ability to reduce systemic inflammation and atherosclerotic lesions, both of which are independent predictors of hypertension (177). However, SCFAs have been implicated in causing damage to the organs affected by hypertension in mice infused with angiotensin II, indicating their role in hypertensive organ damage (178). As a result, a lot of evidence points to the gut microbial community’s influence on blood pressure regulation in the host, with SCFAs functioning as one of the microbial components that contribute to vasomotor tone and blood pressure regulation. Recent research has revealed more evidence that SCFAs play a role in a variety of CVD processes, including ischemia-reperfusion injury, heart repair after myocardial infarction, and arterial compliance impairment (179, 180).

### Bile acid (BA) association with CVD and therapeutics

Bile acids are produced in the liver through the breakdown of cholesterol, are crucial in controlling the absorption of lipids. Primary and secondary BAs can be identified based on their structural features. BAs can also be classed as bound or free based on whether they are conjugated with glycine or taurine (181). In a healthy adult, the liver produces primary bile acids at a daily rate of 500 mg that constitute approximately 72.5% of the total bile acid pool; chenodeoxycholic acid comprises 35%, while cholic acid constitutes 37.5% (182, 183). The synthesis of bile acids occurs through two distinct pathways: the classic (or neutral) pathway and the alternative (or acidic) pathway, each regulated by a specific enzyme. Cholesterol 7-hydroxylase (CYP7A1) enzyme responsible for the classic pathway, whereas oxysterol 7-hydroxylase (CYP7B1) is involved in the alternative pathway (184). Bile acids are stored in the gallbladder and released during digestion into the small intestine. The primary role of bile acids is to emulsify dietary fats and fat-soluble vitamins, facilitating their absorption and transport in the digestive system. Primary bile acids released into the duodenum have a critical function in emulsifying food components and vitamins that are lipid-soluble, enabling their digestion and absorption (185). Secondary bile acids are created when bacterial enzymes change the primary bile acids, which make up roughly 27.5% of bile acid (186).

However, Deoxycholic acid accounts for 25% of the overall bile acid pool, while lithocholic acid and ursodeoxycholic acid collectively make up 2.5% of these bile acids. In a healthy individual, almost 95% of BAs are efficiently reabsorbed in the distal ileum, primarily due to the process of enterohepatic circulation (187). The bile acids that are reabsorbed in the distal ileum are then transported back to the liver to build an effective recycling process. Surprisingly, bile acids, which make up to 2-4 grams of the body’s total weight and play a crucial function, are controlled by a relatively tiny pool (188). This process happens multiple times a day, typically ranging from 5 to 10 cycles daily (189, 190). In order to prevent bile acids from building up to hepatotoxic levels and to limit their impact on cholesterol metabolism, the size of the bile pool is carefully managed through feedback regulation of bile acid synthesis (191). Bile acids also possess strong microbial activity and serve as signaling molecules, acting as ligands for nuclear receptors, thereby impacting various metabolic processes (192). For instance, Farnesoid X-receptor (FXR) activation leads to the suppression of the cholesterol 7a-hydroxylase enzyme. By regulating this enzyme, FXR helps maintain the balance of bile acid synthesis and contributes to the overall control of cholesterol metabolism (193). The gut microbiota plays a significant role in modifying primary bile acids through bacterial salt hydrolase activity. This enzymatic process involves removing the 2OH groups from primary bile acids, transforming them into secondary bile acids (194). Bacteria can lessen BA toxicity by increasing their solubility, giving the gut microbiota a way to defend itself. Additionally, the gut microbiota might change bile acids further before they return to the liver for reconjugation and rejoin the circulation (195). Bile acids serve as a crucial pathway for cholesterol elimination through excretion in feces helping to decrease circulating cholesterol levels and reduce the risk of plaque accumulation (193). However, alterations in the gut microbiome can influence the bile acid synthesis rate, potentially leading to increased plasma levels of LDL cholesterol and an elevated risk of atherosclerosis (196). Thus, maintaining a healthy gut microbiome is essential for regulating bile acid metabolism and its impact on cholesterol levels and cardiovascular health. Additionally, microbial metabolites such as tryptophan and indole have been identified to have significant roles in the development of cardiovascular diseases.

### Therapeutic approaches to gut microbiome

**Figure 6:**
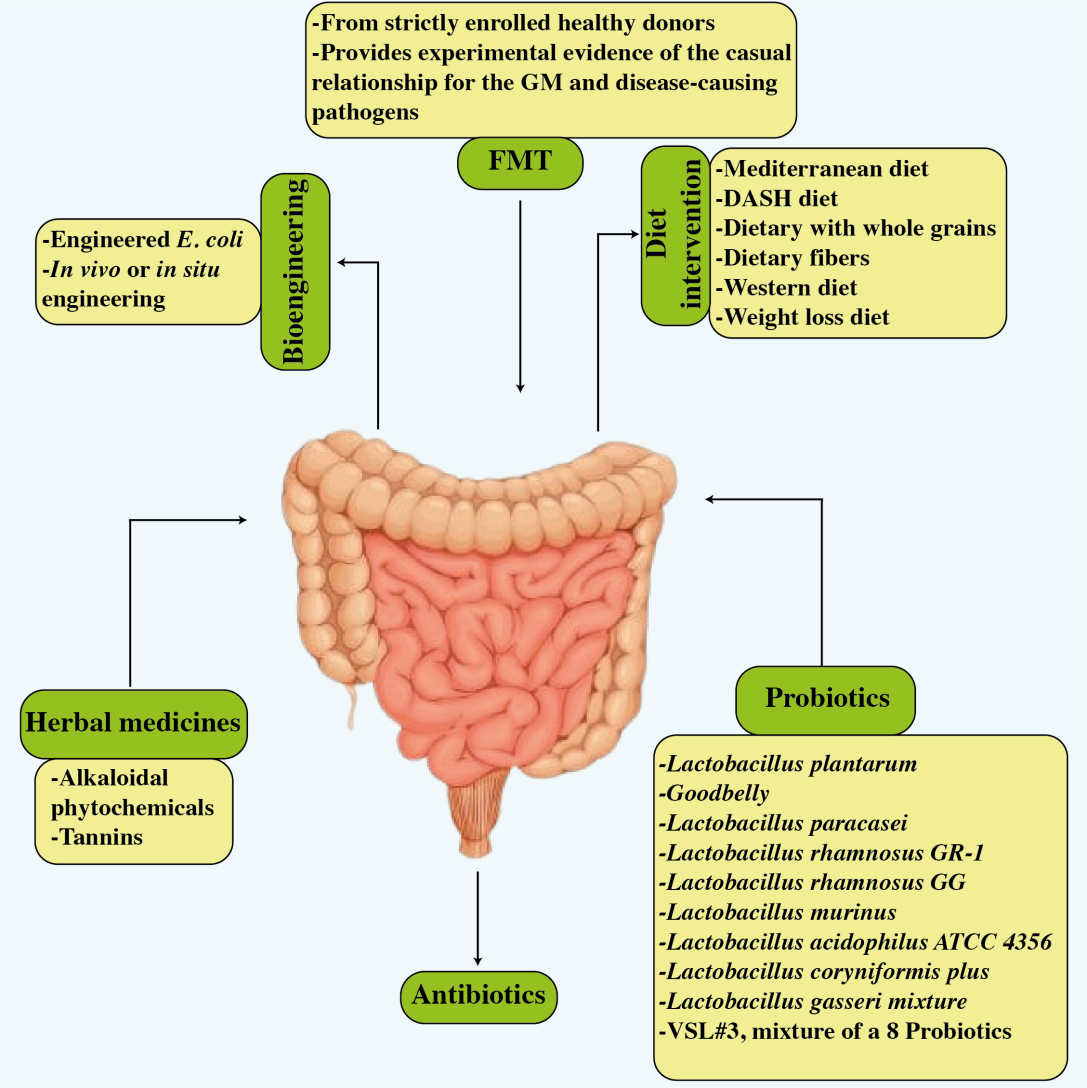
Potential treatments related to improved cardiovascular disease results and improved gut microbiota. The figure illustrates six strategies, i.e., dietary modifications, probiotics, antibiotics, FMT, bioengineering, and herbal treatment.

**Figure 7:**
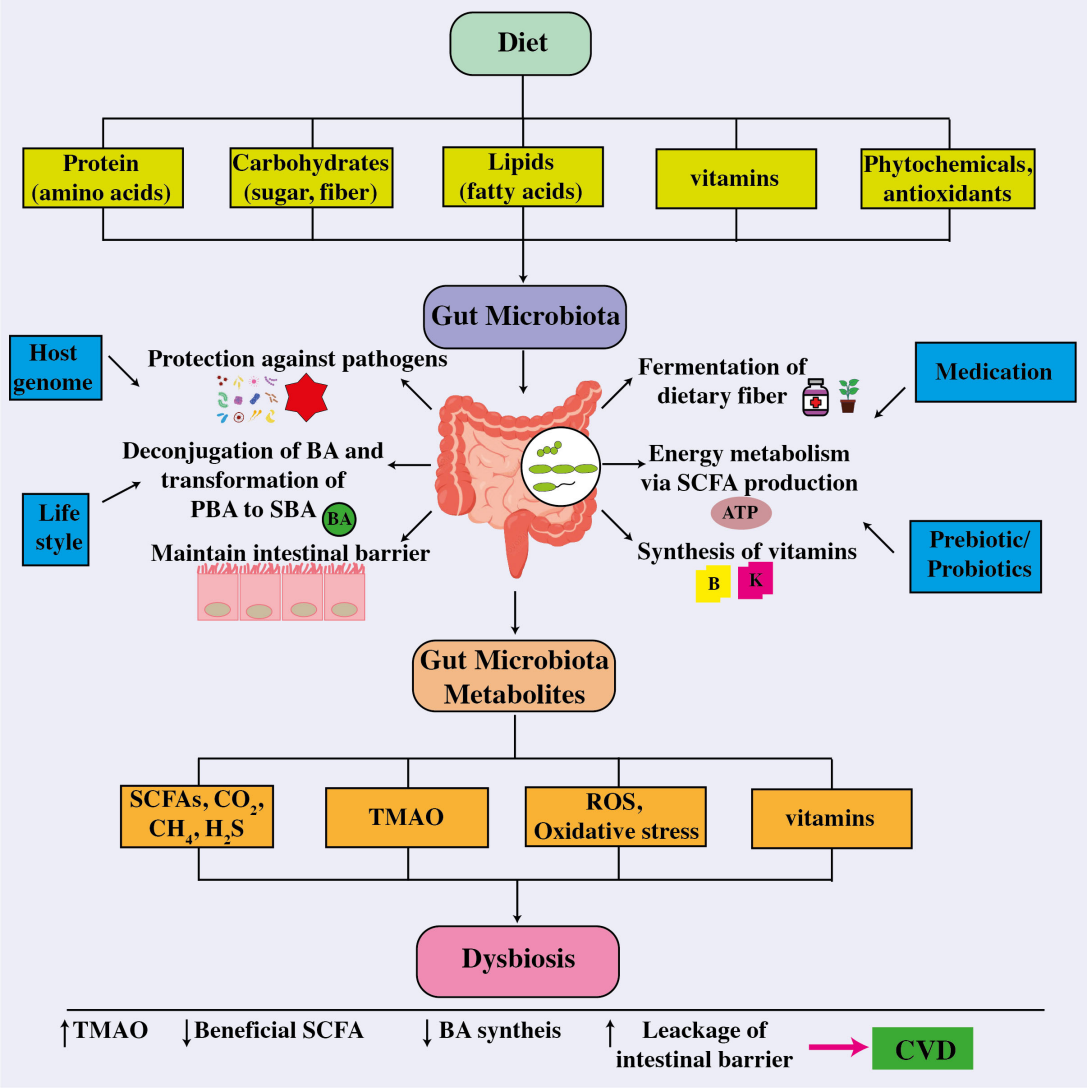
The correlation between the intestinal microbiome, metabolites, and cardiovascular disease. The intricate connection between dietary components absorbed and other factors influencing the gut microbiota, whose composition then influences their functionality and metabolite production and release, a disruption which leads to dysbiosis, thereby affecting host health and the onset and cause of different cardiovascular disorders. ATP, adenosine triphosphate; BA, bile acid; CO_2_, carbon dioxide; CH_4_, methane; CVD, cardiovascular disease; H_2_S, hydrogen sulfide; PBA, primary bile acid; ROS, reactive oxygen species; SBA, secondary bile acid; SCFA, short-chain fatty acids; TMAO, trimethylamine-*N*-oxide.

The novel research implies that the gut microbiota plays a critical role in the progression of cardiovascular illnesses. Therapeutic techniques for influencing the composition and metabolic activity of the gut microbiota have been developed. As shown in **Figure 6**, these options include dietary changes, the use of probiotics and prebiotics, antibiotic treatments, and even fecal transplantation. Notably, these therapies have shown the potential to improve blood pressure control, restore lipid profiles to normal levels, and reduce body weight in people with cardiovascular disease (33). In a vicious cycle, the intricate interaction between dietary components and other variables affects the gut microbiota and pathogenesis of many cardiovascular diseases as shown in **Figure 7** (168).

### Dietary inventions

Numerous scientific studies have provided persuasive evidence supporting the idea that dietary interventions can significantly decrease the risk of cardiovascular problems (197, 198). Diets that frequently occur in Western industrialized societies that feature high consumption of red meat or animal proteins, saturated fats, and simple carbohydrates have been associated with an increased risk of CVD (199, 200). An increasing mass of evidence refers to the intestinal microbiota as a possible avenue for CVD treatment. Current clinical trials on microbe targeting for CVD therapy are summarized in **Table 2** (39). Conversely, the composition of our diet can influence the structure and functioning of the gut microbiome (201). The gut microbiota is greatly influenced by essential food elements such as macronutrients, fiber, polyphenols, prebiotics, and probiotics, which also have an impact on the production and release of major gut microbiome metabolites including SCFAs (202). In a prior study, it was found that diets rich in fiber promote the growth of beneficial symbiotic microbes while preventing the spread of known infectious diseases (203). Moreover, the consumption of a high-fiber diet increased acetate-producing microbiota, which was associated with lower blood pressure and a reduction in cardiac hypertrophy and fibrosis (204). The mediterranean diet, which consists of a high intake of vegetables, fruits, grains, and legumes combined with a low intake of red meat and processed carbohydrates, has been shown to be effective in the prevention of CVD (205). This is mostly due to the high levels of antioxidants, nitrates, and fiber in this diet, and to the low levels of saturated/Trans fatty acids, salt, and phosphate. These elements are expected to reduce inflammation and oxidative stress, promote antioxidant activity, increase nitric oxide bioavailability, and microbiota modulation to improve vascular and cardiac function (206). The Western diet, compared to the Mediterranean diet, is known to raise CVD risk by lowering gut microbiota diversity and beneficial bacteria such as *Bifidobacterium* (207). A study involving mice fed a Western diet, the findings revealed higher plasma concentrations of TMAO and the development of cardiac dysfunction and heart fibrosis (208). The expression of pro-inflammatory cytokines (IL-10) and tumor necrosis factor (TNF-α) as well as interleukin-1 (IL-1), both of which are indicative of increased inflammation, was shown to be altered (208). In a study involving 153 volunteers from four cities in Italy, researchers found that the consumption of fruits, vegetables, and legumes is consistent with the Mediterranean diet led to an increase in fecal SCFA levels (209). This effect is due to fermentation by a greater abundance of bacteria from the *Firmicutes* and *Bacteroidetes* groups (209).

**Table 2:** Clinical trials targeting the gut bacteria in the treatment of cardiovascular diseases (39).

Models	Intervention	Result	Clinical ID
Diet			
Overweight/obese individuals	Mediterranean diet	Positive	NCT03071718
Patients with CAD	Moderate Alcohol Consumption	Positive	No report
Patients with CAD	Calorie restriction	Positive	IRCT20121028011288N15
Patients with CAD	Lacto-Ovo-Vegetarian Diet	Positive	NCT02942628
Overweight/obese individuals	Dietary fibers	Positive	NCT01719900
Patients with CAD	Vegan Diet or the American Heart Association-Recommended Diet	Positive	NCT 02,135,939
Patients with T2D	Dietary fibers	Positive	No report
Patients with heart failure	DASH diet	Positive	No report
Obese hypertensive patients	hypocaloric diet supplemented with probiotic cheese	Positive	ISRCTN76271778
Probiotics			
Subjects with metabolic syndrome	A. soehngenii	Positive	NTR-NL6630
Patients with heart failure	Saccharomyces boulardii	Negative	NCT02637167
Patients with CAD	Lactobacillus rhamnosus GG (LGG)	Positive	IRCT20121028011288N15
Patients with MI	Lactobacillus Rhamnosus G	Positive	IRCT20121028011288N15

	Inulin		
Patients with MI	Lactobacillus rhamnosus capsules	Positive	IRCT20121028011288N15
Overweight/obese insulin-resistant volunteers	A. muciniphila	Positive	NCT02637115
Patients with stable CAD	Lactobacillus plantarum 299v	Positive	NCT01952834
Patients with heart failure	Saccharomyces boulardii	Positive	NCT01500343
Subjects with high-normal blood pressure and mild hypertension	Lactobacillus helveticus	Positive	No report
Probiotics and Prebiotics			
Patients with CAD	Lactobacillus Rhamnosus G and Inulin	Positive	IRCT20180712040438N4
Healthy overweight or obese individuals	Polydextrose and *Bifidobacterium animalis subsp*	Positive	NCT01978691
Prebiotics			
Overweight to obese men	Inulin	Positive	NCT02009670
Children with overweight or obesity	Oligofructose-enriched inulin	Positive	NCT02125955
Mildly hypercholesterolemic individuals	β-glucan	Positive	NCT01408719
Obese women	Inulin-type fructans©	Positive	NCT00616057
Exercise			
Patients with CAD	Bicycle ergometer	Negative	NCT01495091
Patients with CAD	Exercise stress testing	Negative	NCT01495091
Overweight participants	High-intensity interval training	Positive	ACTRN12617000472370
Drug			
Patients with T2D	Berberine and probiotics	Positive	NCT02861261
Patients undergoing elective coronary angiography	broad-spectrum antibiotics	Positive	No report
Patients admitted with acute MI or unstable angina	Amoxicillin, metronidazole	Positive	No report
FMT			
Hypertensive patients	Washed microbiota transplantation	Positive	No report
Obese patients	FMT capsules	Positive	NCT02741518
Patients with metabolic syndrome	Vegan FMT	Positive	NTR 4338
Patients with metabolic syndrome	FMT	Positive	NTR1776

ATP, adenosine triphosphate; BA, bile acid; CO_2_, carbon dioxide; CH_4_, methane; CVD, cardiovascular disease; H_2_S, hydrogen sulfide; PBA, primary bile acid; ROS, reactive oxygen species; SBA, secondary bile acid; SCFA, short-chain fatty acids; TMAO, trimethylamine-*N*-oxide.

Following a mediterranean diet leads to reduced TMAO levels, thereby helping to prevent cardiovascular issues and heart failure (210, 211). A particular study revealed that incorporating ginger supplements into the diet influenced the gut microbiota composition, resulting in a notable rise in fatty acid metabolism (212). Moreover, the presence of miRNAs within ginger-derived exosome-like nanoparticles has the potential to modify bacterial gene expression, influencing the host genome (213). Currently, there is an obvious association between nutrition and intestinal microbes, resulting in varied gut bacterial communities across various diets and geographic locations. However, there is still a huge gap in our understanding of how our food influences the gut microbiome and the impact of the gut microbiota on general host health. More study is needed since nutrition is a low-cost, easy to manage strategy for the possible prevention, control, and management of cardiovascular disease.

### Prebiotics and probiotics

The human colon is filled with probiotics, which are primarily made up of *bifidobacteria* and *lactobacilli*, and are essential for maintaining healthy immune systems, colon microflora, and the production of healthy compounds and also have ability to stop the spread of cancer, lower cholesterol, increase the synthesis of vital cytokines and vitamins, and prevent infections (214, 215). According to Gibson and Roberfroid, prebiotics are defined as non-digestible poly or oligosaccharides that have a positive impact on the host by selectively promoting the growth or activity of specific beneficial bacteria in the colon (216). The *Lactobacillus Plantarum* led to an improvement in the diversity of gut microbial flora, this consumption was linked to a reduction in the incidence of CVD incidents (217). Another study showed by Naruszewicz et al. (218), involving 36 healthy volunteers who were active smokers, revealed that there was a negative correlation between the intake of *Lactobacillus Plantarum* and various health markers, including blood pressure levels, fibrinogen levels, monocyte adhesion, and proinflammatory cytokine levels. These findings suggest that *Lactobacillus Plantarum* may have potential in the primary prevention of atherosclerosis. The normal or moderately elevated cholesterol levels in females experienced low LDL after consuming fermented milk containing *Lactobacillus acidophilus* and *Bifidobacterium longum* (219).

A recent study conducted by Catry et al.(220) revealed a 15-day supplementation of inulin-type fructans (ITFs) had a positive impact on endothelial function in the arteries of n-3 PUFA-depleted ApoE^-/-^ mice. The improvement in endothelial function might be attributed to an increase in bacteria capable of producing nitric oxide, as it helps dilate blood vessels and improve blood flow, ultimately benefiting cardiovascular health. The findings highlight the ITFs potential in promoting cardiovascular well-being, particularly in the context of n-3 PUFA-depleted conditions (221). Similarly, a comprehensive analysis revealed that intake of isolated triterpene fraction yields favorable results on LDL cholesterol levels in the human (222). In addition to ITFs, beta-glucan supplements have also shown the capacity to lower total and LDL cholesterol levels while boosting endothelial vascular reactivity in people in the great health (223). It is crucial to recognize that prebiotics are formed up of a diverse range of chemicals that are controlled by different gut flora (224–227). A fiber-rich diet has been demonstrated to alter the gut microbiota by increasing acetate-producing bacteria, resulting in reduced gut dysbiosis and cardiovascular protection, most notably the transcription factor Egr1, are related to acetate regulation and govern CVD through inflammation, heart fibrosis, and hypertrophy (204).

Another prebiotic, beta-glucan was demonstrated to influence cholesterol levels and glucose homeostasis. A 2-month study that included a beta-glucan dietary plan indicated a significant reduction in LDL and total cholesterol levels. The endothelial function improved in healthy people, showing cardioprotective effects. These effects are mostly due to the production of beneficial SCFA by the gut flora (228). In animal tests, arabinoxylans showed potential as a possible prebiotic. It was discovered that their role in encouraging the growth of *bifidobacteria* and the production of propionate reduces cholesterol and fat deposition (206). Dietary arabinoxylan oligosaccharides raised bacterial populations and butyrate levels in stools in individuals (229). Probiotics help to improve human metabolism by boosting digestive enzyme output, suppressing bacterial enzyme activity, and lowering ammonia generation. *Lactobacillus* and *Bifidobacterium* have beneficial effects on intestinal barrier function and play a protective role in inflammatory diseases by modulating inflammatory and proinflammatory cytokines, which may potentially delay or improve CVD (230, 231). *Akkermansia muciniphila* is also renowned for its probiotic features, and it is related to glucose, insulin, and leptin, all of which have roles in the metabolism of lipids and glucose (232). However, Lactobacillus plantarum efficiently lowered LDL-C and total cholesterol levels while also inhibiting the formation of atherosclerotic plaques in hypercholesterolaemic individuals (233). The preceding research concentrated on the effects of prebiotics and probiotics on cardiovascular risk factors such as inflammation and hypertension, as well as impacts on glucose and lipid metabolism, rather than the direct benefit of atherosclerosis. However, considering their beneficial effect on several CVD risk variables, more research into how these medications affect the onset and progression of CVD is essential.

### Antibiotics

In the regulation of host health, the gut microbiome has a huge impact on the host as a result of antibiotic usage. The use of antibiotics may damage the host’s health in a variety of methods, both directly and indirectly. This impact can alter a variety of bodily processes, including immunological control, metabolism, and ultimately general health (48, 234–237). A variety of antibiotics have shown evidence of affecting blood pressure and intestinal flora. A prime example is the drug minocycline, which has been studied for its capacity to alter the nature of the gut microbiota and control blood pressure (BP) in hypertensive rats (140). Using erythromycin, tetracycline, or doxycycline within the previous five years did not reduce the chance of developing a first MI, according to another population-based trial, and its authors disputed their efficacy in avoiding primary coronary heart disease (CHD) (238). Macrolides antibiotics, such as azithromycin, erythromycin, and clarithromycin, comprise a significant class of orally active antibiotics that function as bacteriostatic agents. In 2013, 51.5 million drugs for azithromycin were prescribed in the United States (239). The use of macrolide antibiotics is believed to increase the risk of cardiovascular diseases such as myocardial infarction (MI), ventricular tachyarrhythmias, and sudden cardiac death (SCD) (240, 241). Further, quadruple antibiotic therapy was shown to significantly lower high systolic and diastolic blood pressures in salt-induced hypertensive rats (242). It helps to realize the research on the effect of antibiotics on blood pressure regulation produced varying outcomes (243).

For instance, minocycline and vancomycin medication in rats led to lower *Firmicutes* levels in the gut, resulting in lower blood pressure in hypertensive rats. It’s noteworthy to note that identical antibiotic therapy actually raised blood pressure in salt-sensitive rats. This difference underlines the intricacy of the gut microbiota-blood pressure interaction and the significance of taking into account a variety of variables that may affect the results of such mediations (244). In a study by Rune et al., they found that ampicillin had the ability to lower mice’s levels of LDL and VLDL cholesterol. Atherosclerosis risk is associated with these forms of cholesterol. As a result, the mice’s aortic atherosclerotic lesions were reduced in size (245). Although a few studies provide promising results, indicating possible benefits in this regard, the efficiency of antibiotics for offering preventive benefits against CVD in trials involving patients is yet unknown (246). However, certain analyses have failed to demonstrate a distinct and obvious link between the use of antibiotics and protection against CVD, yielding unclear outcomes. As a result, more research and analysis are needed to determine whether antibiotics can significantly reduce the risk of cardiovascular disease (240). Furthermore, universal antibiotics can have a variety of impacts on the body, making methods of treating CVD with antibiotics contentious. While certain studies have suggested that taking antibiotics to treat CVD may have some benefits, their broad action can have a number of adverse reactions. Thus, any possible benefits of using antibiotics in treating CVD must be carefully balanced against any dangers and adverse effects that could result from their use. Before making antibiotics a common therapy choice, more study is required to better understand their precise mechanisms of action and potential advantages in the management of CVD.

### Fecal microbiota transplantation as a prevention strategy

Fecal microbiota transplantation (FMT) is a therapeutic method intended to restore a healthy balance of gut microbiota in a recipient (247), by transferring fecal matter from a donor who is in a healthy condition (248). It gained a lot of attention for its safety and efficacy in therapeutic applications after being extensively studied in an array of mammalian species (249).The more complicated nature of propagating gut bacteria compared to those that inhabit the mouth cavity is one of the difficulties that FMT still challenges (250). The FMT involves transferring fecal matter from an adult donor to a recipient with an unbalanced intestinal microbiota and the fecal matter is rich in various microbial populations such as *Clostridioides difficile* (251, 252).This transplantation has shown promising results in treating several intestinal and other chronic diseases and has been researched as a viable therapeutic alternative in clinical applications (253). Notably, FMT has confirmed effectiveness in treating various conditions, including recurrent *Clostridium difficile* infection (254), inflammatory bowel disease (255), and irritable bowel syndrome (256). New research has explored the potential of FMT as a promising approach for addressing cardiometabolic disorders (257).

In 2013, the US Food and Drug Administration (FDA) granted its initial approval for FMT, specifically for managing recurrent *Clostridium difficile* infection. Since then, FMT has gained recognition as a therapy for a wide range of gastrointestinal as well as non-gastrointestinal conditions. However, there remains limited understanding of its mechanism of action and potential long-term side effects (258). The probable therapeutic effects of FMT have also been demonstrated in a number of animal models involving people with severe multiple sclerosis, autism, multidrug-resistant (MDR) infections, and multiple organ failure in seriously confined people (259–261). Recent findings have indicated a lower abundance of *Clostridia* strains that produce butyrate in the intestines with type 2 diabetes mellitus. Conversely, studies have shown a higher prevalence of non-butyrate-producing *Clostridiales* in these patients by demonstrated that both insulin sensitivity and levels of butyrate-producing intestinal microbiota significantly improved following microbiota transplantation (262). Experiments on mice raised the possibility of a brain-gut-microbiota axis that goes in each direction. Various neurological conditions like anxiety, depression, dementia such as Alzheimer’s, and Parkinson’s disorder are caused by an imbalance in this axis (263, 264).

Recently, Park et al. (265) from Inho University Hospital in Incheon, South Korea, used FMT to treat a 90-year-old woman who had severe CDI and Alzheimer’s dementia. Her fecal microbiota diversity drastically altered after the transplant, and her cognitive abilities significantly improved, according to a comparison of the results from before and after the procedure. The study also demonstrated a strong correlation between gut flora and cognitive function. Segal et al. (266) conducted distinct clinical research at Soroka University Medical Centre in Israel with six individuals suffering from both Parkinson’s disease and constipation. These patients were given treatment that included Fecal Microbiota Transplantation (FMT). Moreover, Doll et al.(267) used transplantation of fecal microbiota as add on therapy in two patients with major depression. After 4 weeks, both patient signs of depression improved, and study suggested that FMT be tested extensively for MDD treatment. It was found that transferring feces microbiota from healthy rats with normal heart rates to rats with naturally elevated levels produced positive results. The results included lower systolic blood pressure, enhanced blood vessel functionality, lower levels of oxidative stress and inflammation within blood vessels, and a more favorable balance between two unique types of immune cells, Th17 and Tregs (126, 268). However, the curative benefits of FMT can be linked to a broader variety of bacteria, viruses, fungi, and archaea that can engraft into the recipient host and increase the functional variety of a microbiota FMT is also being examined in almost 300 clinical trials for a variety of disease indications, including autoimmune diseases, neurological difficulties, cancer, host disease, and metabolic and gastrointestinal disorders. There is currently insufficient data to support the relevance of fecal microbiome transplantation about gut microbiota in human patients with CVD, necessitating more research in this field. Different approaches and processing variations, such as donor selection and testing, fecal microbiome transplantation via the upper gastrointestinal tract, enema, or colonoscopy, as well as short- and long-term patient monitoring for adverse effects and treatment efficacy, introduce new challenges to be investigated.

### Exercise

Physical inactivity holds substantial significance as a risk factor for a range of metabolic disorders, and roughly 1/3 of world’s population contributes in inadequate levels of physical activity, which has implications for health (269). Statistics indicate that roughly 3.2million deaths annually can be attributed to inadequate levels of physical activity (270), with healthcare expenses amounting to $117 billion yearly, attributed to conditions resulting from a lack of exercise (271). People who adopt a sedentary lifestyle and fail to engage in regular physical activity are more prone to the development of cardiovascular disease (272), and who are less active face a 30-50% higher risk of developing high blood pressure (273). The researchers predicted that lack of exercise was responsible for 12% of myocardial infarctions (MI), a risk proportion that fell within high blood pressure (18%), CVD cases (6%) and diabetic mellitus (10%) recognized risk factors for heart disease whose incidence is also inversely related to physical activity levels (274, 275). The study confirmed that exercise can enrich the microflora diversity; improve the F/M ratio, which may contribute to weight loss, obesity-related pathologies, and gastrointestinal disorders; and stimulate the proliferation of bacteria, which can modulate mucosal immunity and maintain homeostasis (276–278). Research has demonstrated that exercise has the capacity to increase the levels of the bacterial metabolite known as butyrate (279).While human research in this area is limited, data from several laboratories, including our own, indicate that exercise training can exert a noteworthy influence on the gut microbiota in animal models (280–282). Moreover, the modifications in the gut microbiota brought about by exercise are linked to changes in the host’s physiology, such changes include metabolic rate modifications (283), immunity (280), and even behavior (281). Certainly, exercise training has been demonstrated to increase the concentrations of short-chain fatty acids derived from the gut microbiota in mouse models (284), comprising of two to six carbon atoms, play a crucial role as an energy source for various tissues and are associated with beneficial effects such as reducing inflammation (285), improving insulin sensitivity (286), and inducing the morphology of the central nervous system (287). Notably, levels of LPS are elevated in cardiovascular disease and specific cardio metabolic disorders (288). However, high-endurance training has been shown to have the potential to decrease plasma LPS levels. This suggests that exercise may have a positive impact on reducing inflammation associated with CVD and related metabolic conditions (289). A crucial observation to highlight is that the advantages provided by the gut microbiota due to exercise training were not enduring. This emphasizes the necessity for consistent and regular exercise to sustain a constructive impact on the gut microbiota and the associated health benefits (279). The findings highlighted the broad-ranging benefits of influencing the gut microbiota via physical exercise, stretching beyond the realm of cardiovascular health. Nonetheless, it’s important to recognize that substantial and enduring advantages necessitate prolonged periods and higher-intensity aerobic training. Participating in more extended and intense exercise sessions seems to be pivotal in achieving lasting enhancements in gut microbiota composition and the correlated health advantages for the individual. Consistently adhering to such exercise routines is pivotal for maximizing the influence on the gut microbiota and overall well-being (290). To protect the heart and arteries, physical activity can increase insulin sensitivity, reduce plasma dyslipidemia, proper raise blood pressure, decrease blood viscosity, promote endothelial nitric oxide generation, and improve leptin sensitivity. Furthermore, the preventive impact of exercise on the body involves not only laboratory animal models but also clinical studies, as proven by WHO recommendations (291). Numerous studies have illustrated a clear dose-response correlation between physical activity levels and a decreased incidence of CVD and characterized by reductions in factors such as blood pressure, body weight, oxidized low-density lipoprotein (ox-LDL), and improved glucose tolerance as physical activity increases (292, 293). Although it’s known that exercise protects against CVD by reducing sympathetic impulses, arterial pressure, and heart rate, increasing blood flow and endothelial NO production, causing vessel dilation, and decreasing inflammatory cytokines and oxygen radical formation, the precise processes that lead to transcriptional factor modifications are unknown. Future research could focus on the mechanisms of exercise’s protective effects on the heart and arteries.

## Conclusions

The human intestine is the habitat of the most enormous and varied population of microbes. The main purpose of the gut microbes is to prevent the expansion of potentially lethal germs. However, there is a rising acknowledgment of the intestinal microbiota as a risk variable for developing cardiovascular disease (CVD). Metabolites derived from the gut microbiota, such as short-chain fatty acids, trimethylamine-N-oxide, bile acids, and polyphenols, are critical in maintaining normal cardiovascular function. When these metabolites are out of balance, it has the potential to contribute to an outbreak of CVD. Variations in the composition and diversity of the gut microbiota, known as dysbiosis, have been associated with disorders such as heart failure, atherosclerosis, hypertension, myocardial fibrosis, myocardial infarction, and coronary artery disease. However, the specific mechanisms behind these relationships are still unknown. As a result, the microbiota and its metabolites have emerged as a novel therapeutic target for both CVD prevention and treatment. Ongoing attempts are being made to widen the application of microbiota therapies not only for CVD but also for a variety of other human disorders. Innovations in genomic and metabolomic technology have enabled improved characterization and molecular research of bacteria and their metabolites. Individual microbiome may be profiled in the future utilizing metabolomic/biomarker analysis to measure individual health, potentially delivering specific guidance on food and lifestyle changes. Dietary treatments, the use of pre/probiotics and antibiotics, FMT, TMAO reduction, and regular exercise are current strategies for regulating gut bacteria to improve cardiac function. Further research on microbe-microbe and microbe-host associations may explain how specific metabolites affect the disease process. Improving our understanding of the complex interplay between gut microbiota, host characteristics, and therapeutic response is critical for developing breakthrough precision therapies for cardiovascular disease.

## Author contributions

AL: Writing – original draft. AH: Writing – review & editing. MU: Writing – review & editing. SN: Writing – review & editing. MehrajU: Writing – review & editing. LZ: Writing – review & editing. AU: Writing – review & editing. KU: Writing – review & editing. WA: Writing – review & editing. GW: Writing – review & editing, Funding acquisition, Supervision.
